# Supplementary material for: Small extracellular vesicles containing arginase-1 suppress T-cell responses and promote tumor growth in ovarian carcinoma
Source: Nat Commun. 2019 Jul 5;10:3000. doi: 10.1038/s41467-019-10979-3 (PMC6611910; doi:10.1038/s41467-019-10979-3)
Supplement: Supplementary file 1 — Supplementary Information [file 41467_2019_10979_MOESM1_ESM.pdf]

Small extracellular vesicles containing arginase 1 suppress T-cell responses and promote tumor growth in ovarian carcinoma

Malgorzata Czystowska-Kuzmicz, et al.

Supplementary Table 1: Clinicopathological characteristics of the OvCa patients (all diagnosed with high-grade serous adenocarcinoma) and NC enrolled in the study evaluating arginase expression in primary OvCa by immunohistochemistry (IHC). IHC was performed in 84 samples

	FREQUENCY	PERCENT
**MENOPAUSAL STATUS**		
pre-menopausal	29	33.3
post menopausal	58	66.7
TOTAL	87	100.0
**FIGO-stage**		
I (IA, IB, IC)	37	42.5
II (IIA, IIB, IIC)	10	11.5
III-IV (IIA, IIIB, IIIC, IV)	40	45.0
TOTAL	87	100.0
**DIAGNOSIS**		
serous	40	46.0
endometroid	29	33.3
clear cell	6	6.9
transitional	4	4.6
mucinous	4	4.6
undifferentiated	4	4.6
TOTAL	87	100.0
**GRADING**		
G1	5	5.7
G2	13	15.0
G3	69	79.3
TOTAL	87	100.0
**CYTOREDUCTION**		
optimal	34	39.0
non-optimal	53	61.0
TOTAL	87	100.0
**RECURRENCE**		
no recurrence	19	21.8
recurrence	61	70.1
no remission	7	8.1
TOTAL	87	100.0
**VITAL STATUS**		
alive	34	39.1
dead	52	59.8
unknown	1	1.1
TOTAL	87	100.0

Supplementary Table 2: Results of ARG1 levels evaluation with immunohistochemistry (n = 84) shown as percentages of cells of different staining intensities and the calculated ARG scores[1]

Pat No	ARG 3+	ARG 2+	ARG 1+	ARG neg	ARG score
**1**	70	30	10	0	280
**2**	80	20	0	0	280
**3**	80	20	0	0	280
**4**	40	60	0	0	240
**5**	40	50	10	0	230
**6**	20	70	10	0	210
**7**	20	60	20	0	200
**8**	0	100	0	0	200
**9**	0	100	0	0	200
**10**	20	60	20	0	200
**11**	10	70	20	0	190
**12**	10	60	30	0	180
**13**	0	80	20	0	180
**14**	0	80	20	0	180
**15**	0	70	30	0	170
**16**	0	70	30	0	170
**17**	0	70	30	10	170
**18**	0	70	30	0	170
**19**	0	80	10	0	170
**20**	0	70	30	0	170
**21**	0	70	30	10	170
**22**	0	70	30	0	170
**23**	0	60	40	0	160
**24**	0	60	40	10	160
**25**	0	60	40	0	160
**26**	0	60	40	0	160
**27**	0	70	20	10	160
**28**	0	60	30	10	150

Pat No	ARG 3+	ARG 2+	ARG 1+	ARG neg	ARG score
**29**	0	60	30	10	150
**31**	0	60	30	0	150
**35**	0	30	70	0	130
**36**	10	20	60	10	130
**37**	0	30	70	0	130
**38**	0	20	80	0	120
**39**	0	30	60	10	120
**40**	0	20	80	0	120
**41**	0	20	80	0	120
**42**	0	20	80	0	120
**43**	0	30	60	10	120
**44**	0	20	70	10	110
**45**	0	20	70	0	110
**46**	0	20	70	10	110
**47**	0	30	40	30	100
**48**	0	0	100	0	100
**49**	0	0	100	0	100
**50**	0	0	100	0	100
**51**	0	0	100	0	100
**52**	0	0	100	0	100
**53**	0	10	80	10	100
**54**	0	0	100	0	100
**55**	0	20	60	20	100
**56**	0	0	100	0	100
**57**	0	0	100	0	100
**58**	0	20	60	20	100
**59**	0	10	70	20	90
**60**	0	10	70	20	90
**61**	0	10	70	10	90
**62**	0	20	40	40	80
**63**	0	0	80	20	80

Pat nr.	ARG 3+	ARG 2+	ARG 1+	ARG neg	ARG score
**64**	0	0	80	20	80
**Pat nr.**	**ARG 3+**	**ARG 2+**	**ARG 1+**	**ARG neg**	**ARG score**
**65**	0	0	80	20	80
**66**	0	0	80	20	80
**69**	0	0	80	20	80
**70**	0	0	80	20	80
**71**	0	0	70	30	70
**72**	0	0	70	30	70
**73**	0	0	70	30	70
**74**	0	0	70	30	70
**75**	0	0	70	30	70
**76**	0	0	60	40	60
**77**	0	0	60	20	60
**78**	0	0	60	40	60
**79**	0	0	60	40	60
**80**	0	0	50	50	50
**81**	0	0	50	50	50
**82**	0	0	50	50	50
**83**	0	0	50	50	50
**84**	0	0	20	80	20
**mean**					**126,79**
**median**					**120**
**SD**					**55,97**
**min**					**20**
**max**					**280**

[1] The staining intensity was independently determined empirically by two qualified pathologists and defined as follows: negative (ARG neg), weak (ARG 1+), moderate (ARG 2+) and strong (ARG 3+). The ARG scores were calculated as follows: ARG score = [% positive cells x 3 (strong intensity)] + [% positive cells x 2 (moderate intensity)] + [% positive cells x 1 (weak intensity)].

Supplementary Table 3: Clinicopathological characteristics of the patients enrolled in the study evaluating arginase activity in plasma (n = 81, all diagnosed with high-grade serous adenocarcinoma)

	FREQUENCY	PERCENT
**OvCa-patients:**		
**MENOPAUSAL STATUS**	18	22.2
pre-menopausal	63	77.8
post menopausal	81	100.0
TOTAL		
**FIGO-stage**		
I (IA, IB, IC)	16	19.8
II (IIA, IIB, IIC)	16	19.8
III-IV (IIA, IIIB, IIIC, IV)	49	60.4
TOTAL	81	100.0
**Normal controls:**		
**MENOPAUSAL STATUS**		
pre-menopausal	3	30.0
post menopausal	7	70.0
TOTAL	10	100.0
**DISEASE**		
uterine fibroids	5	50.0
serous cystadenomas	5	50.0
TOTAL	10	100.0

Supplementary Table 4: Arginase activity [U L^{-1}] measured in plasma of OvCa patients and non-OvCa patients (the negative control, NC) at the time of diagnosis

ARG activity [U L^{-1}]	NC n = 10	Stage I n = 16	Stage II n = 16	Stage III n = 49
**mean**	2.29	5.08	9.27	10.74
**median**	2.43	4.02	7.18	9.56
**SD**	1.24	2.92	5.55	6.31
**min**	0.6	1.98	3.18	2.59
**max**	4.23	12.06	21.17	29.39

Supplementary Table 5: Human ovarian cancer cell lines used in the study

**Cell line**	**type**	**source**
OVCAR-3	ovarian adenocarcinoma	Dr. S. Khlief, NIH, USA
AD10	ovarian adenocarcinoma	Dr. S. Khlief, NIH, USA
A2780	ovarian adenocarcinoma	ATCC
Skov3	ovarian adenocarcinoma	ATCC
CaOv-3	ovarian adenocarcinoma	ATCC
MDAH2774	ovarian adenocarcinoma	ATCC
OvCa-14	ovarian adenocarcinoma	Dr. M. Siedlar, Jagiellonian University Medical College, Cracow, Poland
OVP-10	ovarian adenocarcinoma	Dr. B. Szaniawska, Cell Biology Dept., Maria-Sklodowska-Curie Memorial Cancer Center and Institute of Oncology, Warsaw, Poland

The cell lines were not authenticated.

Supplementary Table 6: Primary and secondary antibodies used in the study, n/a: not available

Primary antibodies						
**Protein**	**Clone**	**Appli-cation**	**Dilution**	**Source**	**Catalog number**	**Lot number**
Human/mouse ARG1	polyclonal	WB	1:2000	GeneTex	GTX109242	42263
Human/mouse ARG2	polyclonal	WB	1:500	Santa Cruz	sc-20151	E2913
Human ARG1	polyclonal	IHC	1:100	Abcam	ab128548	n/a
Human/mouse Tsg-101	polyclonal	WB	1:500	Abcam	ab30871	GR192446-1
Human/mouse CD63	MX-49.129.5	WB	1:400	Abcam	ab193349	LO811
EpCAM	EBA-1	WB	1:300	Santa Cruz	sc-66020	A2711
mouse CD9	polyclonal	WB	1:1000	Bioss	bs-2489R	n/a
Human CD81	M38	WB	1:1000	Invitrogen	10630D	527190
V5	D3H8Q	WB	1:1000	Cell Signalling	13202S	2
Human/mouse β-actin	AC-15	WB	1:10 000	Santa Cruz	A5060	n/a
Human CD4-PerCP/Cy5.5.	OKT4	FC	1:200	Biolegend	300530	B210111
Human CD8-PerCP/Cy5.5	RPA-T8	FC	1:200	Biolegend	301032	B209727
Human EpCAM-PE	9C4	FC	1:200	Biolegend	324206	B161699
Human CD3ε-FITC	HIT3a	FC	1:200	eBiosciences	555339	5357931
Human CD3ζ-PE	6B10.2	FC	1:200	eBiosciences	12-2479	4291757
Mouse CD4-PerCp/Cy5.5	RM4-5	FC	1:200	eBiosciences	45-0042-82	4299174
Mouse CD4-APC	GK1.5	FC	1:200	eBiosciences	17-0041-82	E07037-1638
Mouse CD8α-PerCP/Cy5.5	53-6.7	FC	1:200	eBiosciences	45-0081-82	4291993
Human CD9-FITC	ALB6	FC	1:5	Beckman Coulter	IM1755U	40
Human CD81-APC	JS64	FC	1:5	Beckman Coulter	A87789	23
Human CD63-PE	H5C6	FC	1:5	BD Biosciences	558020	8235819
Human/mouse ARG1-PE	polyclonal	FC	1:300	R&D Systems	IC5868P	ADBB0516121
Mouse CD69-PE-Cy7	H1.2F3	FC	1:100	eBiosciences	25-0691	4291385
Mouse CD11c-eF450	N418	FC	1:200	eBiosciences	48-0114	E01232-1633
Mouse CD3ε-APC	17A2	FC	1:200	eBiosciences	17-0032	4302818
Mouse MHCII-PE-Cy7	M5/114.15.2	FC	1:800	eBiosciences	25-5321-80	4321424
Mouse CD45.2-V500	104	FC	1:000	BD	562129	5107958
V5-FITC	polyclonal	FC	1:200	Abcam	Ab1274	GR263967-1
H2-Kb OVA (SIINFEKL)-PE tetramers		FC	1:50	MBL Intl	T03000	T1708003
**Secondary antibodies**						
TrueBlot Anti-mouse Ig-HRP	polyclonal	WB	1:1000	Rockland	p/n 18-8817-33	37199
TrueBlot Anti-rabbit Ig-HRP	polyclonal	WB	1:1000	Rockland	p/n 18-8816-33	37203
Anti-mouse Ig-HRP	polyclonal	WB	1:10000	Jackson Immunores.	715-035-150	107137
Anti-rabbit Ig-HRP	polyclonal	WB	1:10000	Jackson Immunores.	111-035-003	108097
Anti-rabbit Ig-HRP	polyclonal	IHC	1:1000	Dako Agilent	P 0448	n/a

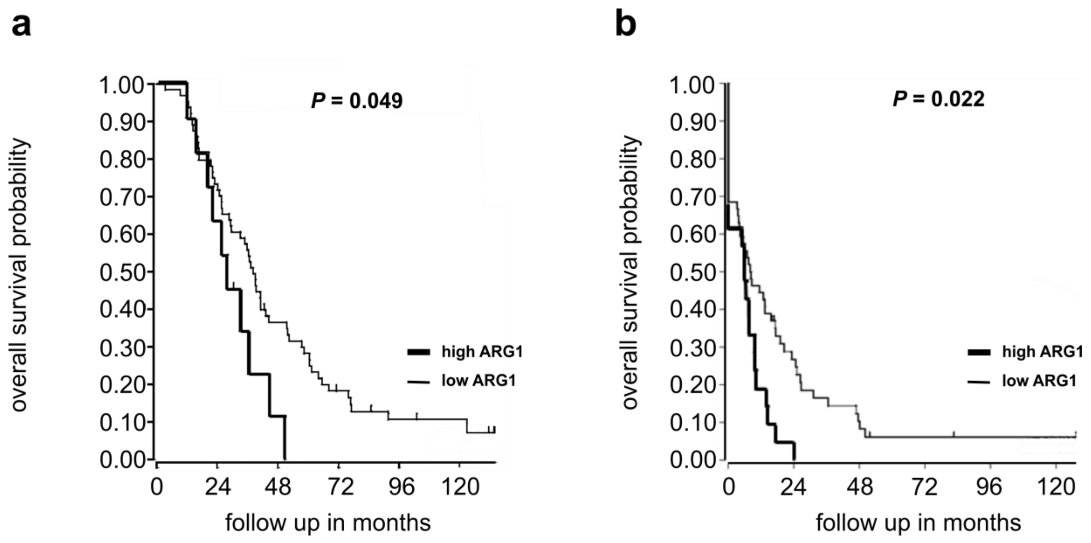

Supplementary figure 1. Transcriptomic analysis of ARG1 expression in patients with OvCa. Kaplan-Meier survival curves of 75 serous OvCa patients from the Pamuła-Piłat-101 MAS5.0-u133p2 data set (GEO accession nr. GSE63885) divided according to ARG1 gene expression. For overall survival the expression cutoff was set at 3.2 and for event free survival at 1.9 by log-rank test. **a** High ARG1 expression (n=11) is associated with a worse overall survival in comparison to low (n=64) ARG1 expression (*P*=0.049). **b** High ARG1 expression (n=21) is associated with a worse event free survival in comparison to low (n=54) ARG1 expression (P=0.022). All data is analyzed in accordance with the public Versteeg database 'R2: microarray analysis and visualization platform' (http://r2.aml.nl).

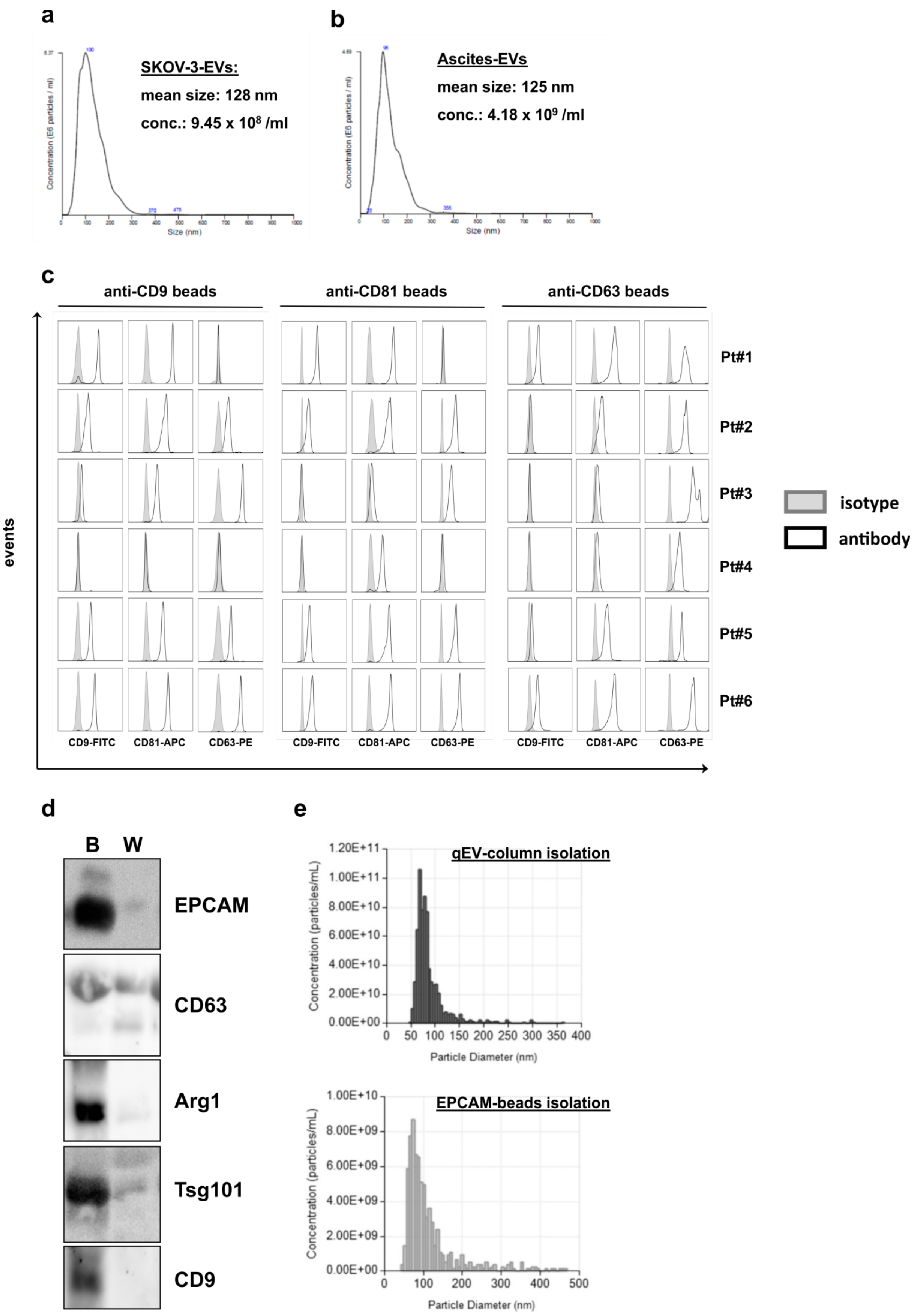

Supplementary Figure 2. Isolation and characterization of OvCa-TEX. Representative example of concentration and size distribution of OvCa EVs isolated

by sequential centrifugation from the supernatant of an OvCa cell line **a** and from patient's ascites **b** as measured by nanoparticle tracking analysis (NTA). **c** The presence of typical exosomal tetraspanins determined by flow cytometry on EVs. Distinct EVs-subtypes were immuno-captured from concentrated total EVs pre-enriched from ascites by sequential centrifugation using beads coated with anti-CD9, anti-CD81 or anti-CD63. **d** EVs were isolated from ascites with anti-EPCAM coated magnetic beads (Miltenyi). Expression of EPCAM, ARG1 and typical exosomal markers (CD63, Tsg101, CD9) in the bead-bound fraction (B) determined by Western blotting. The fraction obtained after column wash (W) was used as control. **e** Size and concentration measurements by Tunable Resistive Pulse Sensing (TRPS: Izon qNano) of EVs isolated from ascites by size-exclusion chromatography using a qEV-column (upper panel) and EPCAM-beads (lower panel).

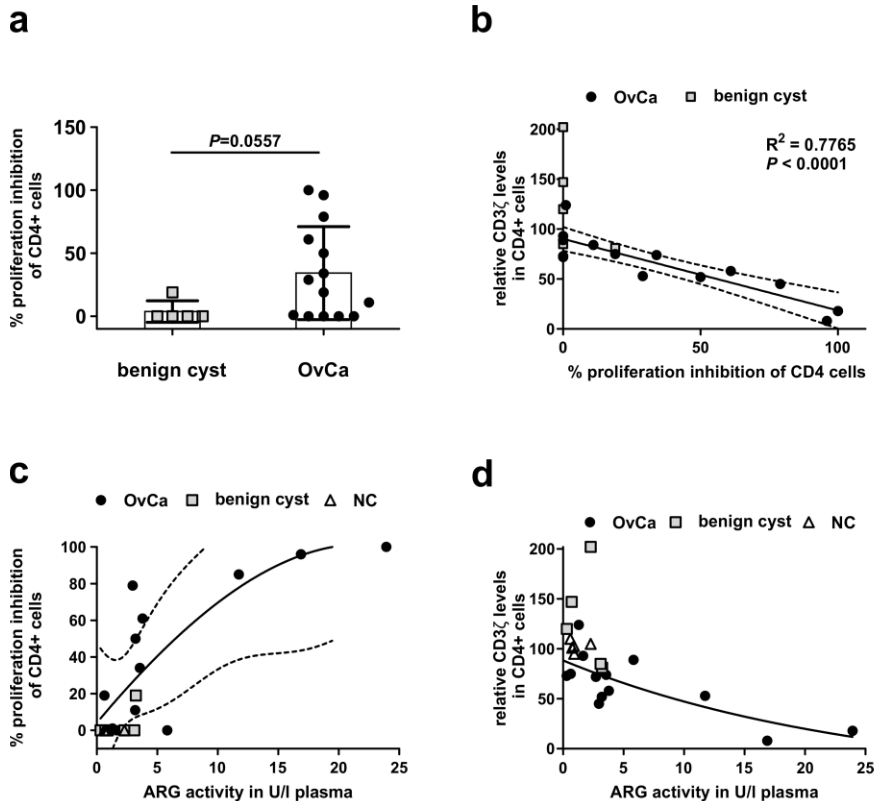

Supplementary Figure 3. EVs-ARG1 inhibit proliferation of $CD4^+$ T-cells. **a** Percent of proliferation inhibition of peripheral blood $CD4^+$ T-cells in n=14 OvCa patients and n=5 patients with benign ovarian cyst normalized to the mean proliferation of n=5 healthy controls. Data show means ± SD, *P* value calculated in Mann-Whitney U test. **b** Correlation between the peripheral blood $CD4^+$ T-cell proliferation inhibition and CD3ζ levels. R^2 value and *P* value (Mann-Whitney U test) were calculated with GraphPad Prism 6.0. Dotted lines mark the 95% confidence intervals. **c** Percentages of peripheral blood $CD4^+$ cells proliferation inhibition as a function of ARG activity in plasma of n=14 OvCa patients and n=5 patients with benign cyst of the ovary. Dotted lines mark 95% confidence intervals. **d** CD3ζ levels evaluated in flow cytometry as a function of ARG activity in the plasma of n=14 OvCa patients and n=5 patients with benign cyst of the ovary.

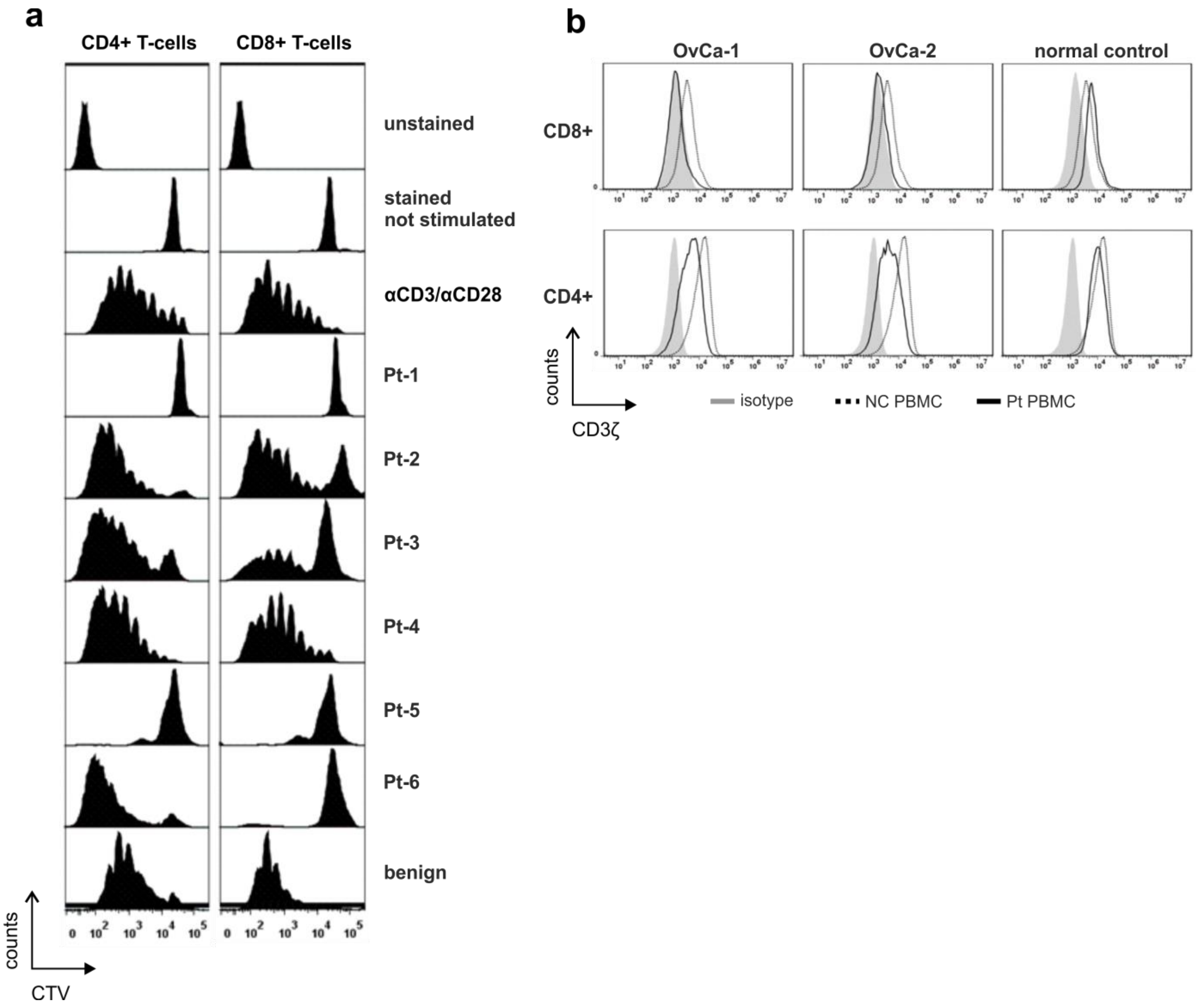

Supplementary Figure 4. Peripheral T-cells of OvCa patients show decrease proliferation capability and CD3ζ expression. **a** Proliferation histograms of the CD4$^+$ (left panel) and CD8$^+$ (right panel) T-cell fraction of PBMCs of 6 representative OvCa patients, showing different degree of proliferation inhibition in comparison to T-cells of a healthy control and of a patient with benign disease. **b** Representative histograms of CD3ζ chain expression in the CD8$^+$ (upper panel) and CD4$^+$ T-cell fraction (lower panel) of PBMCs of two OvCa patients and a patient with benign disease.

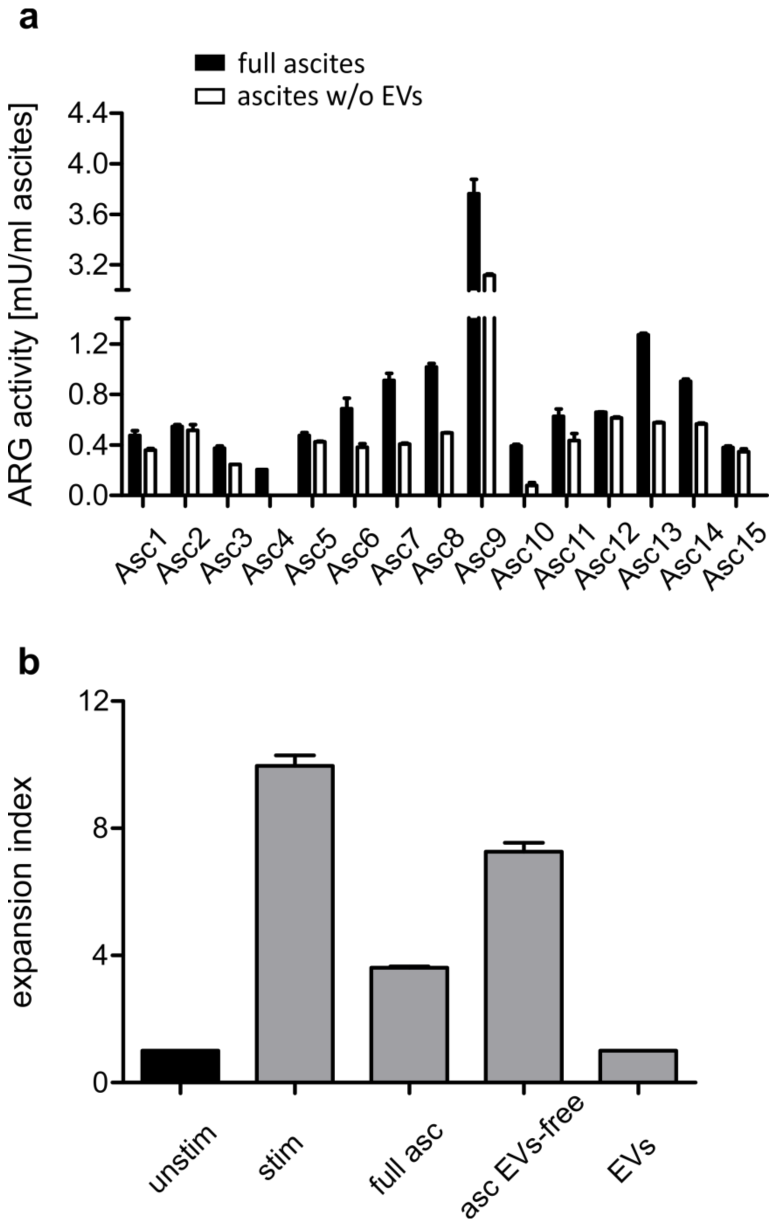

Supplementary Figure 5. Arginase activity and suppressive potential of OvCa ascites. **a** Arginase activity in full ascites or in the ascitic supernatant after removal of EVs by sequential centrifugation. Data show means ± SD from duplicates. **b** Suppression of proliferation of αCD3/αCD28-stimulated $CD8^+$ T-cells cultured for 5 days in full ascites, the ascitic fraction obtained after removal of EVs (both 90% v/v) or in presence of EVs isolated from 2 ml of full ascites. Cell proliferation is expressed as expansion index, i.e. the fold-expansion of the responder cells, calculated by the FlowJow v7.6.5 software. Data show means ± SD from duplicates.

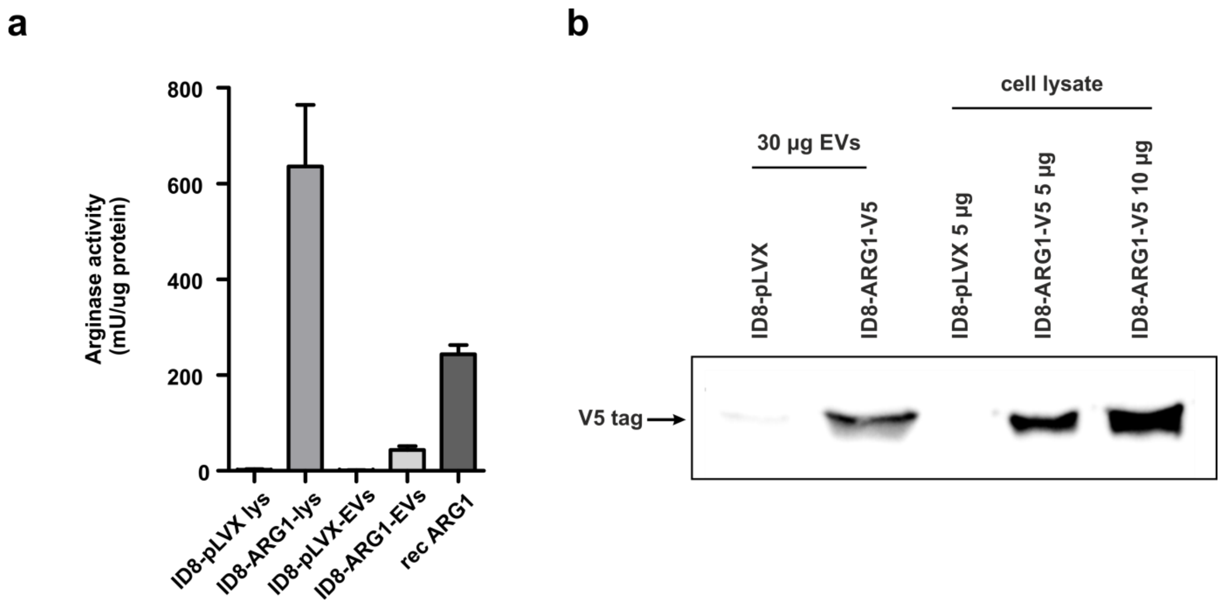

Supplementary Figure 6. Arginase activities and expression levels in ID8 cells. **a** Arginase activity in the lysates of ID8-pLVX and ID8-ARG1-V5 cells and in the corresponding EVs. Mean relative expression of 3 independent experiments (error bars, SD). **b** Immunoblotting for V5-tag in the lysates of ID8-pLVX and ID8-ARG1-V5 cells and the corresponding EVs.

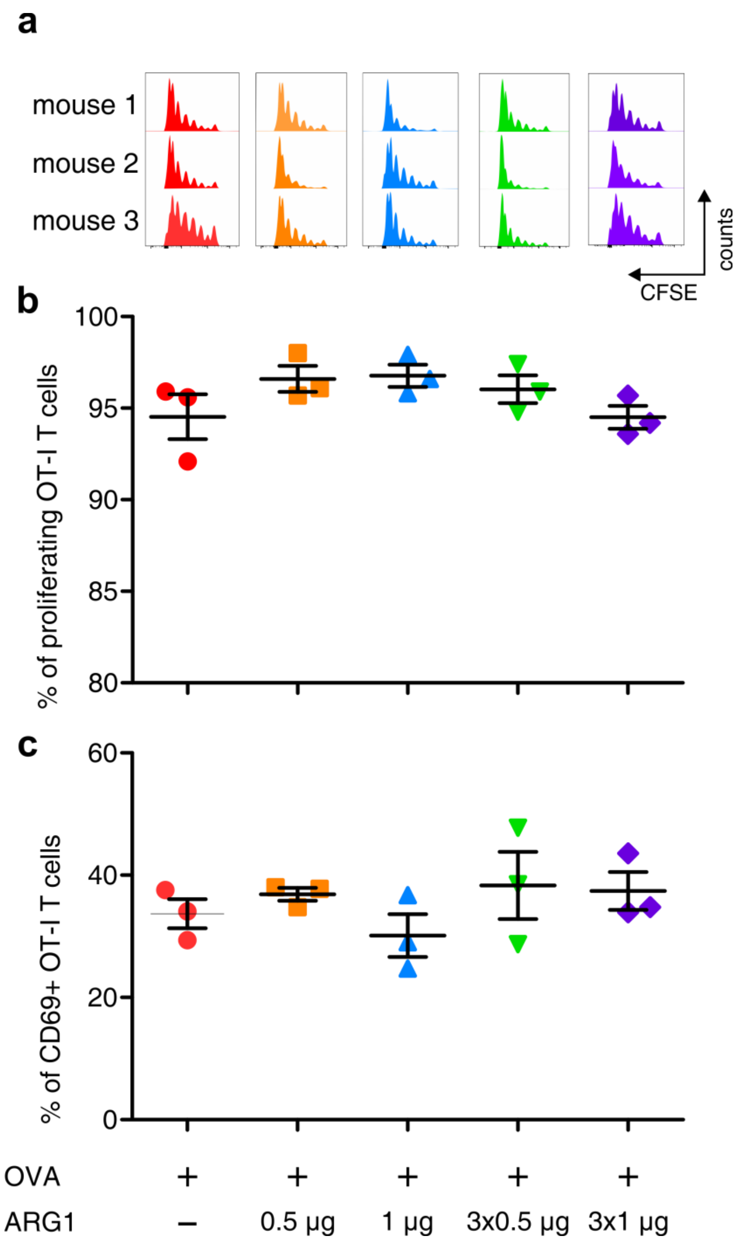

Supplementary Figure 7. Recombinant ARG1 fails to inhibit specific T-cell proliferation *in vivo*. OVA-specific, CFSE-stained CD8$^+$ T-cells (4×10^6 cells) isolated from OT-I mice were injected intravenously into C57BL/6 mice and 24 h after adoptive transfer 5 µg of OVA protein ± different amounts of recombinant murine ARG1 were injected s.c. into the right thigh. In some groups subcutaneous injections of recombinant ARG1 were repeated daily for the next two days. After 72 h CD8$^+$ T-cells were isolated from right inguinal lymph nodes, stained with SIINFEKL-tetramers and analyzed for proliferation by flow cytometry. Representative proliferation profiles **a** and percentages **b** of *in vivo* proliferating OT-I T-cells in the presence of recombinant ARG1 are shown. Data show means ± SD The gate was set on live, CD8$^+$ tetramer-positive T-cells. **c** Percentages of activated (CD69$^+$) OT-I T-cells. Data show means ± SD

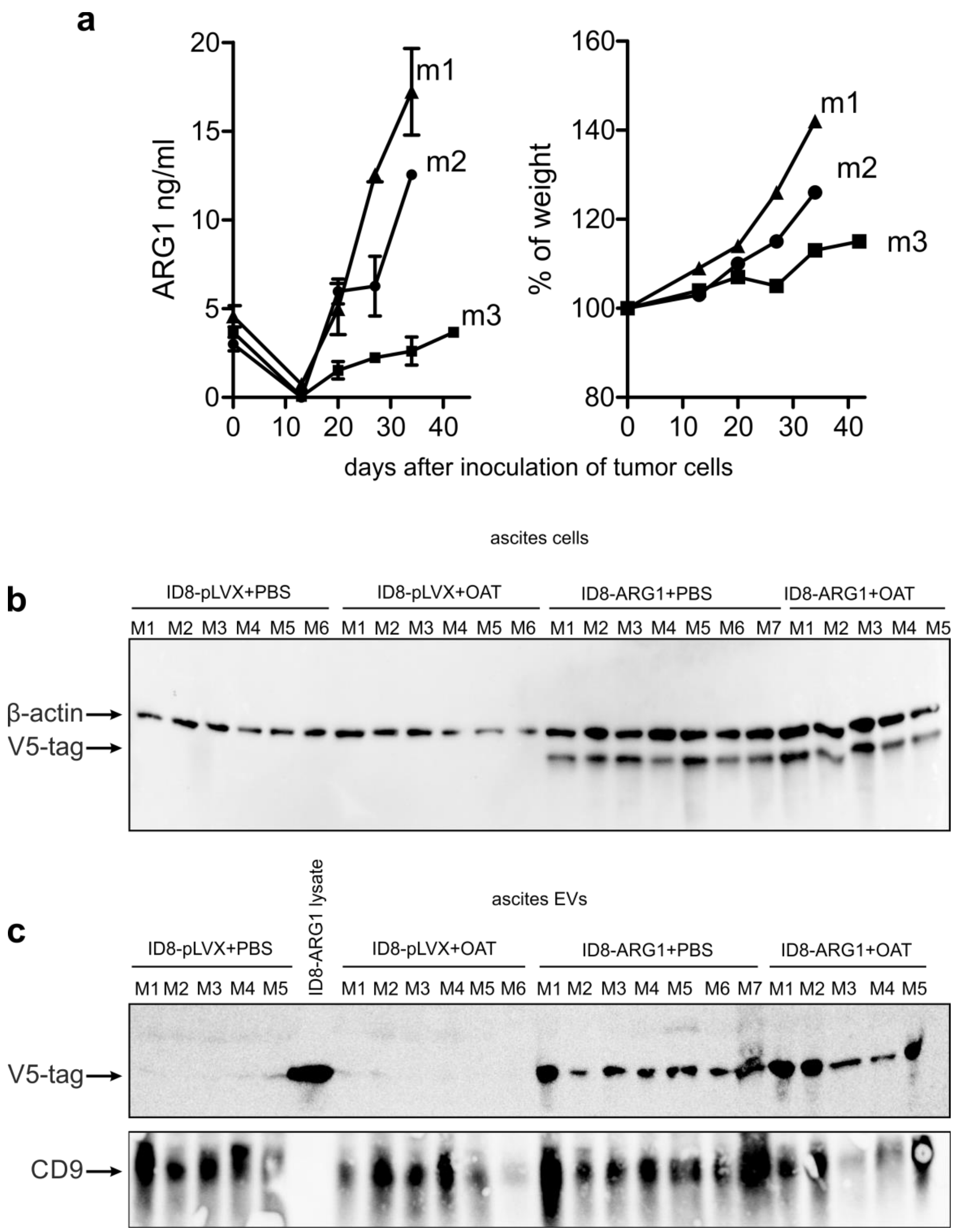

Supplementary Figure 8. ARG1-V5-tag-positive EVs and cells are detectable in ascites of ID8-ARG1 tumor-bearing mice. **a** ARG1 levels (left) in the plasma of 3 mice inoculated i.p. with $4×10^6$ ID8-ARG1 cells measured with ELISA and changes in mouse weight (right) in corresponding mice. **b** Immunoblotting for V5-tag (and β-actin used as a loading control) in cells isolated from the peritoneal cavity of mice inoculated with controls or ARG1-overexpressing ID8 cells. Ascites was collected 28 days after inoculation of tumor cells. Equal amounts of protein (10 µg) were loaded per lane. **c** Immunoblotting for V5-tag (and CD9 used as a marker for the EV preparations) in the EVs isolated from peritoneal cavity. Equal amounts of protein (30 µg) were loaded per lane (except for ID8-ARG1 lysate = 5 µg).

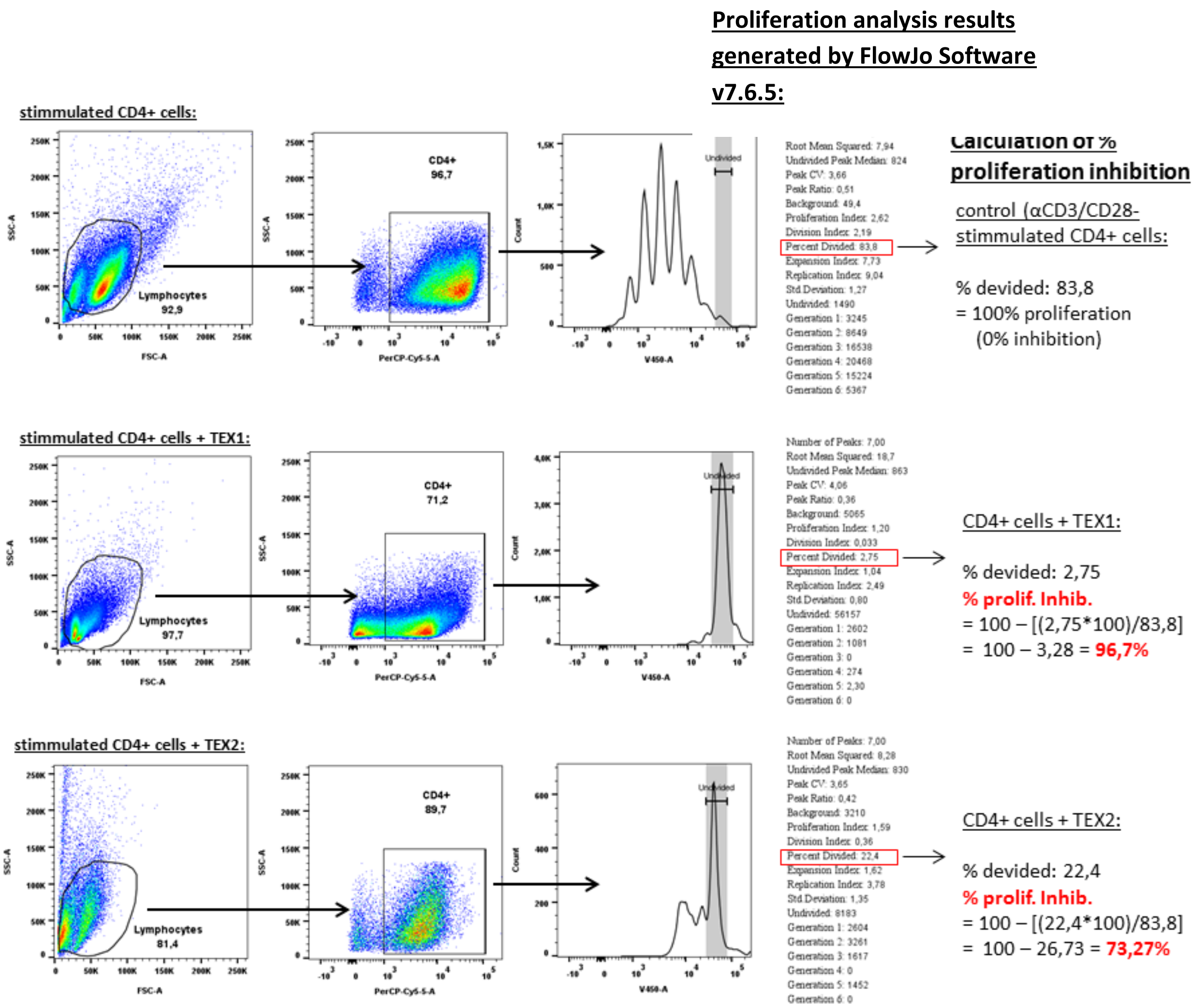

Supplementary Figure 9. Gating strategy used to analyze the data shown in Figure 3c, Figure 3d, Figure 3e, Figure 4, Supplementary Fig 3(a-d), Supplementary Fig 4, Supplementary Fig. 5b and Supplementary Fig. 7. Exemplary gating strategy used for the $CD4^+$ T-cell population (analogous strategy was used for the $CD8^+$ T-cell population). A minimum of 10 000 cells were acquired within the lymphocytes gate. Proliferation histograms were generated in FlowJo v7.6.5. Exemplary calculation of the percentage of proliferation inhibition based on the proliferation analysis in FlowJo v7.6.5.

CD4+ T-cells:

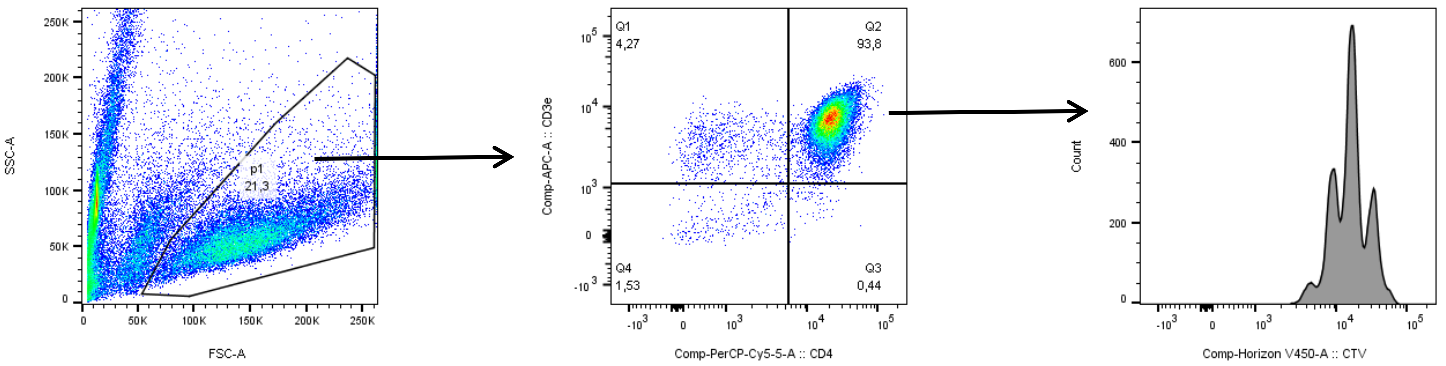

CD8+ T-cells:

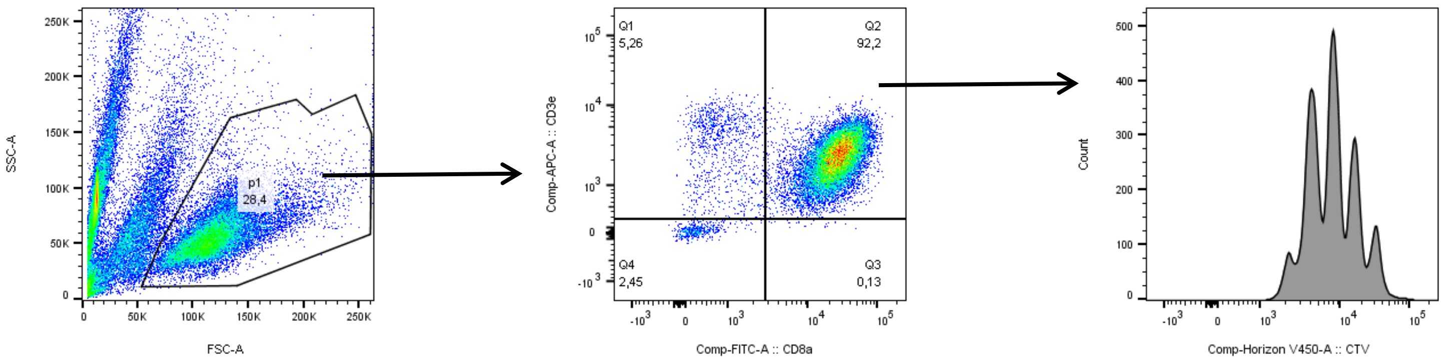

Supplementary Figure 10. Gating strategy used to analyze the data shown in Figure 5. A minimum of 10 000 cells were acquired within the lymphocytes gate. Proliferation histograms were generated in FlowJo v7.6.5.

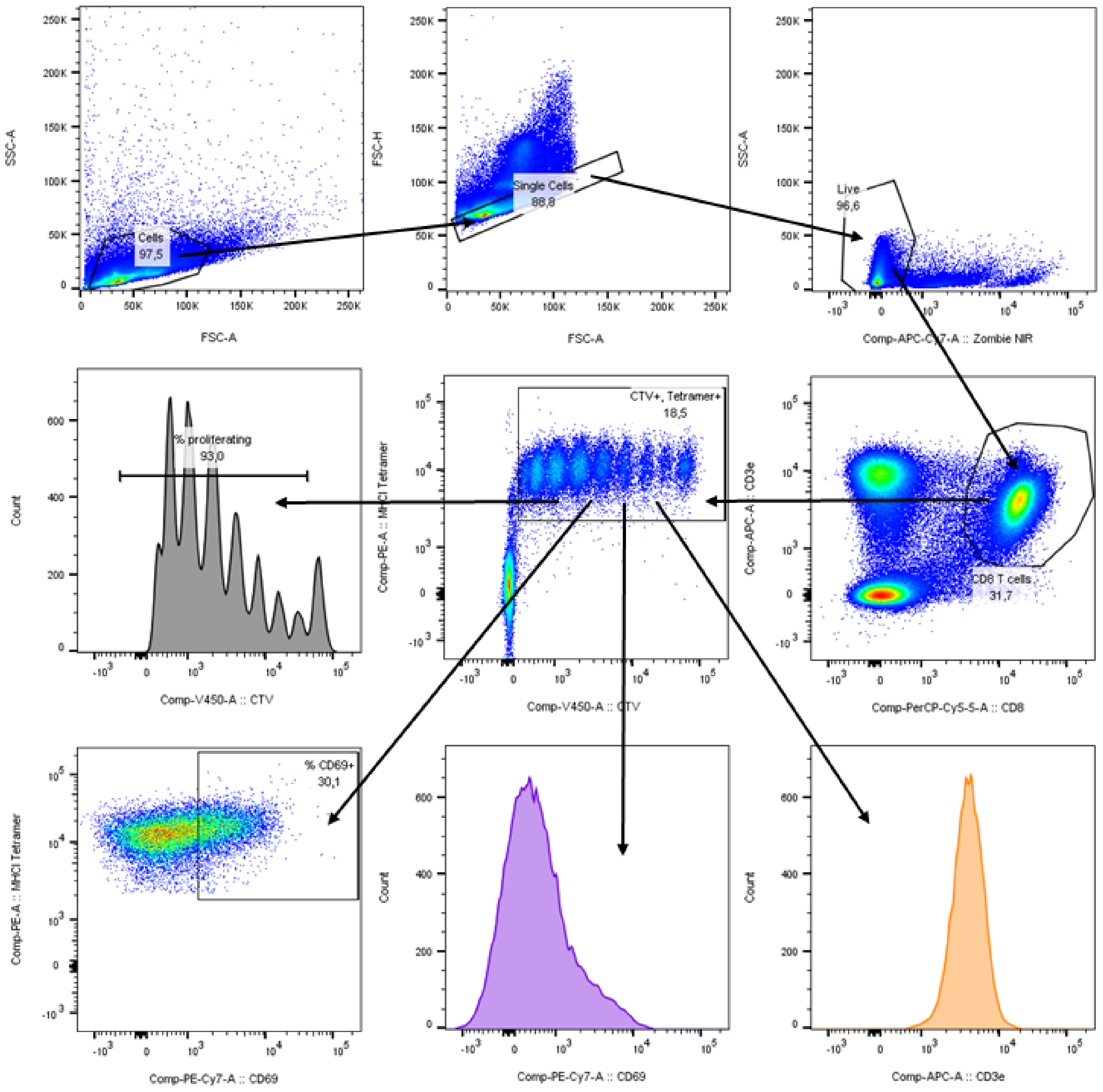

Supplementary Figure 11. Gating strategy used to analyze the data shown in Figure 6. A minimum of 10 000 cells were acquired within the CTV=Tetramer$^+$ gate. The gate for CD69$^+$ cells was set based on the unstimulated control. Proliferation histograms were generated in FlowJo v7.6.5.

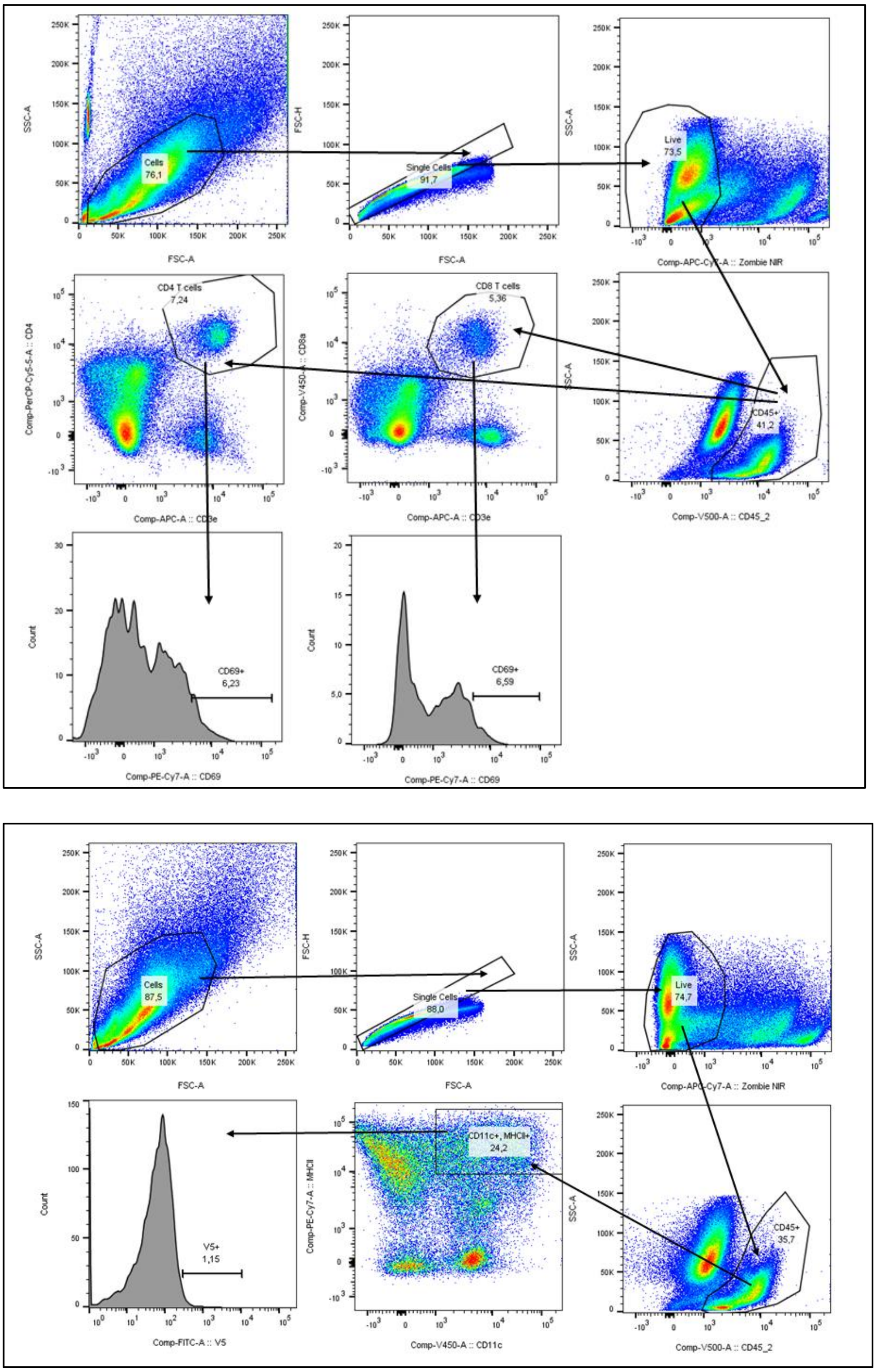

Supplementary Figure 12. Gating strategy used to analyze the data shown in Figure 7b (upper panel) and Figure 7c (lower panel). A minimum of 20 000 cells (Figure 7b) or 50 000 cells (Figure 7c) were acquired within the $CD45^+$ gate. Gates for $CD69^+$ cells and $V5^+$ cells were set based on FMO controls. Proliferation histograms were generated in FlowJo v7.6.5.
